# Regulation of Ferredoxin-NADP^+^ Oxidoreductase to Cyclic Electron Transport in High Salinity Stressed *Pyropia yezoensis*

**DOI:** 10.3389/fpls.2018.01092

**Published:** 2018-07-25

**Authors:** Bin Yu, Jianfeng Niu, Jianhua Feng, Meiling Xu, Xiujun Xie, Wenhui Gu, Shan Gao, Guangce Wang

**Affiliations:** ^1^Key Laboratory of Experimental Marine Biology, Institute of Oceanology, Chinese Academy of Sciences, Qingdao, China; ^2^Laboratory for Marine Biology and Biotechnology, Qingdao National Laboratory for Marine Science and Technology, Qingdao, China; ^3^Center for Ocean Mega-Science, Chinese Academy of Sciences, Qingdao, China; ^4^College of Life Sciences, University of Chinese Academy of Sciences, Beijing, China

**Keywords:** electron transportation, ferredoxin-NADP^+^ reductase, *Pyropia yezoensis*, stress responding, environmental acclimation

## Abstract

*Pyropia yezoensis* can survive the severe water loss that occurs during low tide, making it an ideal species to investigate the acclimation mechanism of intertidal seaweed to special extreme environments. In this study, we determined the effects of high salinity on photosynthesis using increasing salinity around algal tissues. Both electron transport rates, ETR (I) and ETR (II), showed continuous decreases as the salinity increased. However, the difference between these factors remained relatively stable, similar to the control. Inhibitor experiments illustrated that there were at least three different cyclic electron transport pathways. Under conditions of severe salinity, NAD(P)H could be exploited as an endogenous electron donor to reduce the plastoquinone pool in *Py. yezoensis*. Based on these findings, we next examined how these different cyclic electron transport (CETs) pathways were coordinated by cloning the gene (*HM370553*) for ferredoxin-NADP^+^ oxidoreductase (FNR). A phylogenetic tree was constructed, and the evolutionary relationships among different FNRs were evaluated. The results indicated that the *Py. yezoensis* FNR showed a closer relationship with cyanobacterial FNR. The results of both real-time polymerase chain reaction and western blotting showed that the enzyme was upregulated under 90–120‰ salinity. Due to the structure-function correlations in organism, *Py. yezoensis* FNR was proposed to be involved in NAD(P)H-dependent Fd^+^ reduction under severe salinity conditions. Thus, through the connection between different donors bridged by FNR, electrons were channeled toward distinct routes according to the different metabolic demands. This was expected to make the electron transfer in the chloroplasts become more flexible and to contribute greatly to acclimation of *Py. yezoensis* to the extreme variable environments in the intertidal zone.

## Introduction

Photosynthetic organisms do not always grow under optimal conditions. Indeed, higher plants, eukaryotic algae, and cyanobacteria usually suffer from drought or desiccation stress. Many studies have shown that dehydration leads to a decrease in PS II activity and the activation of stress responding process (Baker, 1991; Golding and Johnson, 2003). Under these conditions, the cyclic electron transport (CET) around PS I was enhanced (Müller et al., 2001). The effects of PS I-driven CET and the pseudo-CET on the stress response have been widely accepted (Shikanai and Yamamoto, 2017).

There are several PS I-driven electron transport routes consisting of various redox components in chloroplasts. The common factor of these CETs involves reduction of the plastoquinone (PQ) pool by either ferredoxin (Fd) or NAD(P)H/NADH, followed by re-reduction of P700^+^ by electrons through the cytochrome *b6/f* complex and plastocyanin. The difference among these CET pathways is usually attributed to the enzyme that mediates reduction of PQ from the PS I acceptor side (Bukhov and Carpentier, 2004). Currently, the PGR5-PGRL1 protein-dependent pathway, which shows antimycin A (AA) sensitivity (Shikanai, 2014), and NDH-dependent cyclic flow, which is mediated with the chloroplastic NAD(P)H dehydrogenase complex (NDH-complex), whose activity is inhibited by rotenone (Ro) (Endo et al., 2008), have been extensively studied. In addition, there is another CET termed the rotenone-insensitive NAD(P)H-PQ oxidoreductase pathway, which is sensitive to dicoumarol (INN) and diphenyleneiodonium, but not to Ro (Corneille et al., 1998). The NAD(P)H-PQ oxidoreductase was postulated to be involved in and the enzyme belonged to a flavoprotein, which catalyzes PQ reduction using NADH or NAD(P)H as electron donors (Corneille et al., 1998). Recently, a kind of pseudo-CET mediated by flavodiiron protein was reported to reduce O_2_ to H_2_O directly using electrons from PS I (Yamamoto et al., 2016). Notably, photoproduction of O_2_^-^ around PS I can consume the extra energy absorbed through water-water cycle (Asada, 1999), which constitutes another pseudo-CET and complements the function of PS I-driven electron transport (Shikanai and Yamamoto, 2017).

FNR was initially identified as a chloroplast reductase, catalyzing the electron transfer from reduced Fd to NADP^+^ to produce NAD(P)H during linear electron flow (LEF). In higher plants, FNR has been shown to be expressed as distinct photosynthetic type (leaf type) and non-photosynthetic type (root type) isoproteins. For root FNR, reduced Fd is produced at the expense of NAD(P)H (Shinohara et al., 2017). The reverse reaction from NAD(P)H to reduced Fd mediated by FNR has also been observed in cyanobacteria and some algae. Moreover, in the NDH-dependent CET pathway, FNR was found to be attached to the NAD(P)H dehydrogenase complex and function to recycle electrons from NAD(P)H to PQ and then to cytochrome *b6f* (Endo and Asada, 1996). In *Chlamydomonas reinhardtii*, the identified supercomplex mediating Fd-dependent CET contained FNR (Iwai et al., 2010). Zhang et al. (2001) reported that FNR was copurified with cytochrome *b6f* and inferred that the complex played an important role in Fd-dependent CET. Although the exact role of FNR in different organisms is still unclear, the significance of this molecule in CET is undisputed.

*Pyropia yezoensis* (previously called *Porphyra yezoensis*), belongs to the genus of *Pyropia* (Sutherland et al., 2011) and is a common rhodophyte found in the intertidal zone. At low tide, the algae are exposed to air, and the salt concentration of extracellular water can increase up to 10 times compared with that of seawater (Sutherland et al., 2011). This severe dehydration can lead to water loss of up to 85–95% of the total water content of the algae. However, the algae can fully recover when rehydrated (Blouin et al., 2011). Wiltens et al. (1978) studied the effects of osmotic stress in *Pyropia* through fluorescence induction and found that the fluorescence time-course was similar to that in the presence of 3-(3′,4′-dichlorophenyl)-1,1-dimethylurea (DCMU), which blocks electron transport from PS II to PQ, suggesting that electron flow between the two photosystems was inhibited upon shortage of water. Fork and Öquist (1981) showed that both the fraction of light transfer from PS II to PS I and the energy absorbed by PS I increased in dehydrated *Pyropia*. Thus, the enhancement of PS I was closely correlated with water shortage. However, the mechanisms mediating electron transport in the context of high salinity in this intertidal seaweed are still unclear.

The involvement of FNR in protection of the photosynthetic apparatus has been demonstrated in *C. reinhardtii*, cyanobacteria and the higher plants (Zhang et al., 2001; Hcp et al., 2002; Iwai et al., 2010), although characterization of its significance in the intertidal macro-algae *Py. yezoensis* remains limited. Accordingly, in this study, we analyzed the physiological importance of FNR in hyperosmotic treated *Py. yezoensis*. Our findings provided important insights into the stress response networks of *Py. yezoensis* and the adaptation of this organism to the extreme variable environments of the intertidal zone.

## Materials and Methods

### Algae and Cultivation

Fresh *Py. yezoensis* was randomly collected from the intertidal zones of Qingdao (36°3′0″N, 120°21′57″E) in China. The thalli were rinsed with seawater and cultured in Provasoli’s enriched seawater medium at 15°C with 50-μmol photons m^-2^ s^-1^ of cool-white light in a 12-h light/12-h dark cycle. The medium was bubbled in culture flasks continuously with filter-sterilized air and renewed every day.

### Rapid Light Curves and Chlorophyll Fluorescence Measurement

The photosynthetic parameters of different samples were determined *in vivo* using a Dual-PAM-100 measuring system (Heinz Walz, Effeltrich, Germany). First, based on the methods of Porcía and Matilde (2011), all samples were kept in normal seawater or solutions with different salinities (from 40‰ to 150‰) for 2 h and provided with 50 μmol photons m^-2^ s^-1^ of cool-white light before determination. The samples were then subjected to rapid light curve measurements using a pre-installed software program with nine incremented actinic lights (photosynthetic active radiation [PAR] 11, 18, 27, 58, 100, 131, 221, 344, and 536 μmol photons m^-2^ s^-1^) for 30 s. For each group, at least three data points for the electron transport rate ETR (II) and increasing PAR were exported from WinControl and averaged. With these averaged values, the rapid light curves were fitted according to the model of Eilers and Peeters (1988). The maximal quantum yield of PS II (α), the ETR_max_, and the onset of light saturations (I_k_) were calculated subsequently.

Chlorophyll fluorescence measurement was carried out using automated procedures provided by Dual-PAM software at room temperature. The intrinsic minimum fluorescence (F_0_) of each sample was detected under weak measuring light (12 μmol m^-2^ s^-1^) after keeping the samples in the dark for 10 min. Saturating actinic light pulses (SP, intensity: 6,000 μmol m^-2^ s^-1^, duration: 300 ms, wavelength: 635 nm) were applied to obtain maximum fluorescence (F_m_). Variable fluorescence (F_v_) was measured by estimating the difference between F_m_ and F_0_. Based on the results of rapid light curves, actinic light (51 μmol photons m^-2^ s^-1^) from 635 nm LED arrays was set during the measurement. With repetitive application of saturation pulses, the quantum yields of PS II (YII) were derived by the software. The relative rate of photosynthetic electron transport, which was calculated as the product of quantum yields and PAR, was used to indicate the activity of the photosystem. Due to the absence of a direct CET monitoring method (Shikanai, 2014), we employed the difference between ETR (I) and ETR (II) (*D*-value) to assess the significance of CET. The P700 signal was obtained through determination of the absorption difference between 875 and 830 nm (Klughammer and Schreiber, 1994).

### Inhibitor Treatment and the Effects on the Redox State of PS I

For determination of the possible CET pathways in *Py. yezoensis*, different inhibitors, including Ro, AA, and INN were applied to test the effects on the redox state of PS I under saturation pulse. By adding DCMU, electron transport from PS II to PS I was inhibited. All inhibitors and DCMU were purchased from Sigma (Aldrich, Shanghai, China). The inhibitor stocks were freshly prepared. DCMU and AA were prepared in ethanol. Ro was dissolved in DMSO, and INN was dissolved in water. The final concentrations of the inhibitors were optimized (AA: 40 μM, Ro: 50 μM, INN: 250 μM, and DCMU: 10 μM) according to the report by Corneille et al. (1998). The samples were stressed first with different salinities and then incubated with inhibitors in the dark for 10 min before measurement (Jeanjean et al., 1999). The values of the P700^+^ signals under saturation pulse were collected, and data analysis was performed according to the method reported by Endo and Asada (1996). The normalization equation was as follows: CI = (Ci – min[Ci:Cn]) / (max[Ci:Cn] – min[Ci:Cn]), where Ci and Cn are the raw P700^+^ signals, CI is the corresponding normalized P700^+^ signal at different time points. Each P700^+^ signal curve was derived from the average value of three replicates. In the absence or presence of DCMU, the P700^+^ signals from different salinity stressed samples were compared to illustrate the effects of salinity on CET. The results of treatment with different inhibitors with the same salinity stressed samples were scaled and plotted in the same coordinate system, thus, the effects of inhibitors on the re-reduction of P700^+^ were evaluated. The P700^+^ curve of the algae in DCMU under corresponding salinity was designated as the control.

### Cloning and Sequencing of FNR

Total RNA and genomic DNA were extracted from fresh thalli with TRIzol (Invitrogen, Carlsbad, CA, United States) and a genomic DNA extraction kit (TaKaRa, Dalian, China), according to the manufacturers’ instructions. The cDNA used for sequence amplification was synthesized using a SMART RACE cDNA kit (Clontech Lab. Inc., Mountain View, CA, United States). Based on the conserved domain of FNR inferred from NCBI, degenerate oligonucleotide primers (FNR-anti1: GTNGGNACNCCNARRAANARCCA; FNR-1: AARACNGTNWSNYTNWSNGT. N = A/C/G/T, R = A/G, W = A/T, S = C/G and Y = C/T) were designed. The polymerase chain reaction (PCR) products were recovered and sequenced. According to the sequence information obtained from the above fragment, a specific primer for 3′ RACE was synthesized (FNR-2: GCGACCATCATCATGCTTGCCACG). Another primer (FNR-anti2: GCGACCATCATCATGCTTGCCACG) was designed according to the newly obtained sequences as a nested primer to perform gene walking, yielding the 5′ end of the full-length *FNR* gene, combined with the FNR-anti1 primer. PCR primers (FNR-DS: CCCGGCCCAGTCGGATCGCACACGC; FNR-DA: CCCACAAAGGGCAATAGTAGAAC) were designed based on the full-length cDNA sequence obtained above, and the genomic sequence of the *FNR* gene was amplified and sequenced.

### Sequence and Phylogenetic Analysis

The DNA sequence of FNR was examined using the NetGene2 Server^1^, and its homology to other known sequences was analyzed using the BLAST program, available at the NCBI website^2^. The deduced amino acid sequence from the cDNA was analyzed with the Expert Protein Analysis System^3^, and the conserved domains were predicted using Pfam HMM^4^. The possible transmembrane regions were analyzed with TMpred in the ISREC-Server^5^; only regions with scores above 500 were considered significant transmembrane regions.

Based on the results of BLAST sequencing, several FNRs from different species, including cyanobacteria, chlorella, red algae, and higher plants, were downloaded from GenBank and aligned using the multiple sequence alignment tool. The alignments were used for phylogenetic analyses by MEGA version 4.0 (Tamura et al., 2007). A phylogenetic tree was generated by the neighbor-joining (NJ) method, with 1,000 bootstrap replicates. NJ distance analysis was performed with the Kimura 2-parameter model, and the variation rate among sites was modeled with a gamma distribution (shape parameter = 2), in which all positions containing gaps were ignored in the distance estimation.

### Real-Time PCR Assay

According to the results of PAM, several interesting samples treated with different salinities were collected for quantitative assays using real-time PCR. For real-time PCR assays, the elongation factor1-α (*eEF-1α*) gene was amplified simultaneously as an internal control (Wang et al., 2014). The primer sequences were as follows: *FNR* forward, 5′-GACAAGGTGCACATCACTGG-3′; *FNR* reverse, 5′-AAGCTCGGTTTGGTAGAGCA-3′ (208-bp product); *eEF-1α* forward, 5′-GTCACCAGGCATAACCAT-3′; *eEF-1α* reverse, 5′-GAGGGCGGAAGACATACT-3′ (135-bp product).

Real-time PCR assays were carried out using SYBR Green I Master Mix (TOYOBO, Japan) on an iCycler iQ Real-Time PCR Detection System (Bio-Rad, Hercules, CA, United States). The transcript levels of the *EF-1α* gene were used to normalize the relative transcription of the *FNR* gene in samples stressed with different salinities. The relative fold change of *FNR* transcription was calculated by the 2^-ΔΔ^*^C^*^t^ method (Livak and Schmittgen, 2001).

### Western Blotting Analysis of FNR

According to the deduced amino acid sequence of FNR derived, the possible complementarity-determining regions (CDRs) were screened. Then, the nucleic acid sequences of the selected peptides were synthesized *in vitro* and cloned into an expression vector. Protein immunogens with the grafted CDRs from FNR were overexpressed in *Escherichia coli*, purified by Ni-affinity chromatography and subjected to animal immunization. The mouse with the best immunological response was chosen and its spleen cells were fused with myeloma cells to generate hybridomas. Antibodies secreted by the different clones were then assayed using an ELISA method for their ability to bind to the antigen. The most productive and stable clones were selected and injected in the peritoneal cavity of mice. The mice ascites was then prepared and the monoclonal antibodies were recovered using an Affi-Gel Protein G column according to the manufacturer’s instructions (GE Healthcare, Uppsala, Sweden). The prepared antibody was maintained in 50 mM sodium phosphate buffer (PBS, pH 7.4) for later use.

Thalli were treated for 2 h with the stress conditions described above and were then ground in liquid nitrogen. The soluble proteins were extracted with a high Mg^2+^ medium (30 mM MgCl_2_, 1 mM MnCl_2_, 2 mM ethylenediaminetetraacetic acid, 30 mM KCl, 0.25 mM KH_2_PO_4_, and 50 mM HEPES, pH 7.6). Phenylmethylsulfonyl fluoride was added to a final concentration of 2 mM. Aqueous extracts were collected by centrifugation at 13 000 × *g* at 4°C for 30 min.

The volume of extract was concentrated after normalization according to the results of protein quantitative in each sample and two parallel SDS-PAGE separations were performed. One was stained with Coomassie brilliant blue R-250 and the other was used for western blotting. Western blotting was performed with horseradish peroxidase-coupled secondary antibodies (Thermo Fisher Scientific, Shanghai, China), and the target protein bands were visualized using 3,3′-diaminobenzidine reagent (Pierce, Rockford, IL, United States). The SDS-PAGE gel and the western film were scanned using a GS-800 Calibrated Imaging Densitometer (Bio-Rad, Hercules, CA, United States). Image analysis software Quantity One (Bio-Rad, version 4.6.9) was used to analyze differences in band intensities between the control and the salt-treated samples. To eliminate errors, Gauss Model Tracing was performed according to the software instructions, and a protein band of 19 kDa located in the SDS-PAGE gel was used as the internal control to calibrate the relative bands intensities of FNR on the western blot film. The protein band of 19 kDa, belonging to the overlapping of α and β subunits of R-phycoerythrin. R-phycoerythrin is not only a functional molecule of the antenna pigment protein, but also serves as a nitrogen source reservoir. The high content of this protein in the algae makes it will not fluctuate dramatically and this allow it could be used as the internal control in western blotting.

### Data and Statistical Analysis

During rapid light curves measurements, at least three data points for ETR(II) at increasing PAR were exported from WinControl and averaged for each group. All the photosynthetic parameters were derived from three independent measurements. Statistical analysis was performed using one-way analysis of variance, and the values were deemed to be significantly different when the *P*-values were less than 0.05. Each P700^+^ signal curve was derived from the average value of three replicates, and replicates were normalized from the corresponding P700^+^ signal determination under saturation pulse. The results from real-time PCR were subjected to *t*-test analysis, and results with *P*-values of less than 0.05 were considered statistically significant.

## Results

### Effects of Various Salinities on Photosynthetic Parameters

**FIGURE 1:**
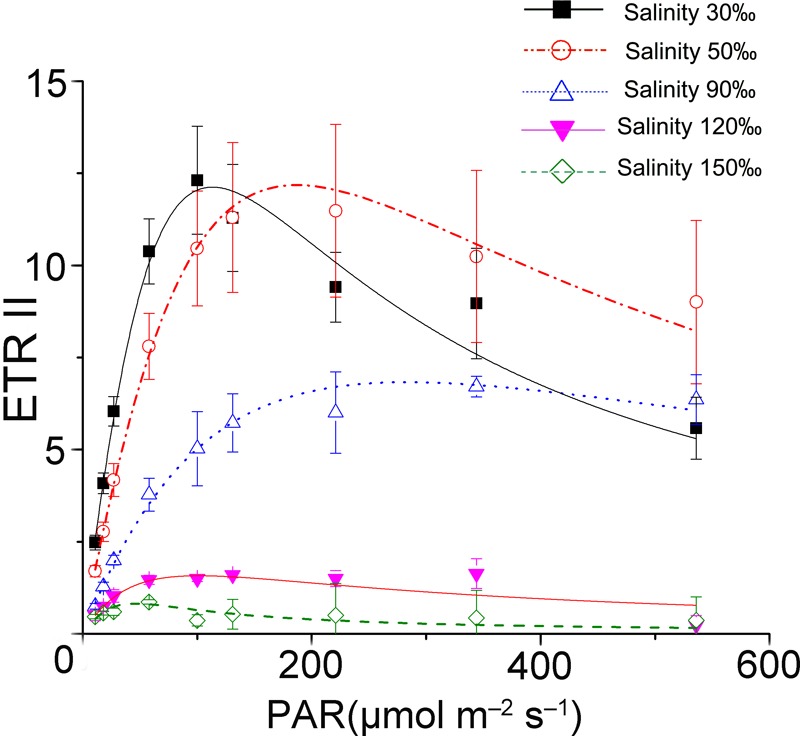
Saturation curves of *Py. yezoensis* under different salinity stresses. The solid lines with filled squares indicate the control; the dashed lines with open circles represent samples stressed with 50‰ salinity; the dotted lines with upward-pointing open triangles indicate the 90‰ salinity-treated algae; the solid lines with downward-pointing filled triangles indicate 120‰ salinity; and the dashed lines with open diamonds indicate 150‰ salinity-treated algae. Three independent determinations were performed, and the average values were analyzed using Origin2015 software to fit the saturation curves according to the following formula: ETR = PAR/(a × PAR^2^ + b × PAR + c).

The results of rapid light curves (**Figure 1**) showed that the salinity stresses decreased the maximal quantum yield of PS II (α). The value of onset of light saturation (I_k_) derived from the fitting curve of the control was about 50 μmol photons m^-2^ s^-1^. Additionally, the I_k_ value increased unexpectedly under mild and moderate high salinity stresses. After reaching the maximum (90 μmol photons m^-2^ s^-1^) at 90‰ salinity, the I_k_ value decreased sharply to a level similar to that of the control at 120‰ salinity. The activity of PS II became negligible when the samples were subjected to treatment with 150‰ salinity.

**FIGURE 2:**
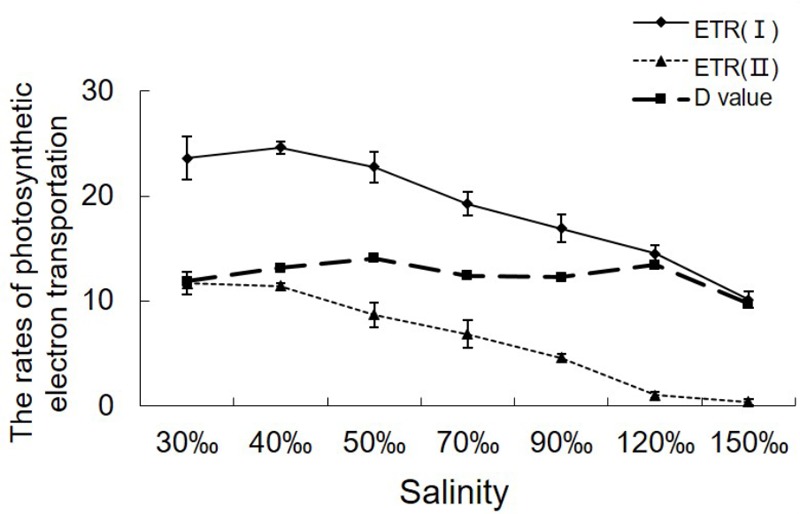
Variations in the relative rates of photosynthetic electron transport in *Py. yezoensis* under different salinities. The ETR data were the means of five independent experiments (±SDs). *D*-values were derived from the differences between ETR (I) and ETR (II).

ETR (II) showed a continual decrease when algal samples were exposed to increasing salinity, reaching zero when the salinity increased to 120‰ (**Figure 2**). Except for a slight increase in ETR (I) under 40‰ salinity (*P* < 0.05), ETR (I) showed a pattern similar to that of ETR (II) in samples treated with salinities ranging from 70 to 150‰ (*P* < 0.01). Despite the obvious reduction in electron supply from PS II, the difference between ETR (I) and ETR (II) remained similar between the control sample and the samples stressed with 120‰ salinity.

### Changes in P700^+^ Signals Under Saturation Pulse

**FIGURE 3:**
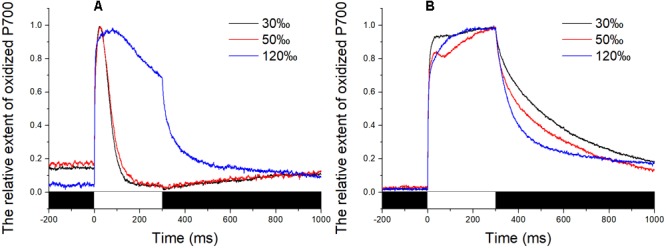
Time course of the P700^+^ signal in *Py. yezoensis* under different salinity stresses. The start point of the saturation pulse (SP) of 6,000 μmol m^-2^ s^-1^ was set as 0, and the duration of the SP was 300 ms. The value of the P700**^+^** signal was normalized before plotting. **(A)** Time course results under different salinities. **(B)** Curve of the samples under DCMU. The bar under the coordinate system indicates the duration of different irradiation conditions. The white indicates the saturation flash and black indicates dark.

The P700^+^ signal reached a high level upon exposure to the saturating pulse for all determinations and then returned to baseline immediately in the control and in thalli treated with 50‰ salinity (**Figure 3A**). In comparison, the decrease in the P700^+^ signal of algae treated with 120‰ salinity seemed lower than those in the control and in samples subjected to mild high salinity stress, although the value went back to baseline when the flash was switched off (**Figure 3A**). Application of DCMU led to a continuous increase in the P700^+^ signal of the samples under illumination with a saturation pulse (**Figure 3B**). Subsequently, the P700^+^ signal showed an obvious enhancement (**Figure 3B**).

**FIGURE 4:**
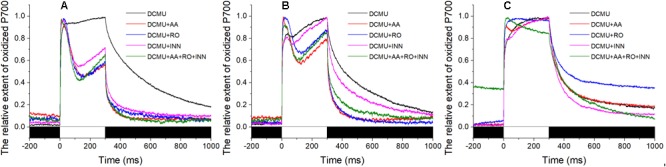
Effects of inhibitors of the P700^+^ signal under different salinity stresses with DCMU. **(A)** Curve of the samples in the normal seawater. **(B)** Curve of the samples stressed with 50‰ salinity. **(C)** Curve of the samples stressed with 120‰ salinity. Inhibitor solutions were prepared with seawater having different salinities and the stock solution. Inhibitor treatment was performed in the dark for 10 min using the *Py. yezoensis* thalli treated with the indicated salinity. The data are the means of three independent experiments (±SDs). C, control; AA, antimycin A; Ro, rotenone; INN, dicoumarol. The bar under the coordinate system indicates the duration of different irradiation conditions. The white indicates the saturation flash and black indicates dark.

In the presence of DCMU, addition of AA inhibited the corresponding portion of the total CET. Unlike the stable maintenance of the redox state of PS I in the sample stressed with 120‰ salinity (**Figure 4C**), the P700^+^ signal showed a slight decrease initially and then an increase during the 300-ms illumination with the saturating flash (**Figures 4A,B**). The extent of the decrease in the P700^+^ signal in samples exposed to 30‰ salinity was larger than that of the samples exposed to 50‰ salinity. However, the slope of the subsequent rise in the P700^+^ signal showed the opposite trends. Under conditions of 50 and 30‰ salinity in the presence of DCMU, the effects of Ro and BHC on the P700^+^ signal showed trends similar to that in samples treated with AA. Importantly, the P700^+^ signals in the BHC, Ro, and AA groups showed gradual increases (**Figures 4A,B**). Under 120‰ salinity, AA led to a slight decrease in the P700^+^ signal, Ro showed no effect, and only a negligible decrease in the P700^+^ signal was detected by BHC treatment (**Figure 4C**).

As described above, the inhibitors had different effects on the P700^+^ signal, demonstrating that there were at least three different CETs in *Py. yezoensis*. We also tested whether the reduction of oxidized PS I caused by the saturation pulse was inhibited by the combination of these three inhibitors. However, the results showed that the variations in P700^+^ signals were similar to those of separate inhibitors when the algae were exposed to 50 or 30‰ salinity (**Figures 4A,B**). In contrast, the combination of AA, Ro, and BHC decreased the P700^+^ signal continuously during the saturation pulse in the presence of DCMU under 120‰ salinity stress (**Figure 4C**).

### Sequence and Phylogenetic Analysis of *Py. yezoensis* FNR

**FIGURE 5:**
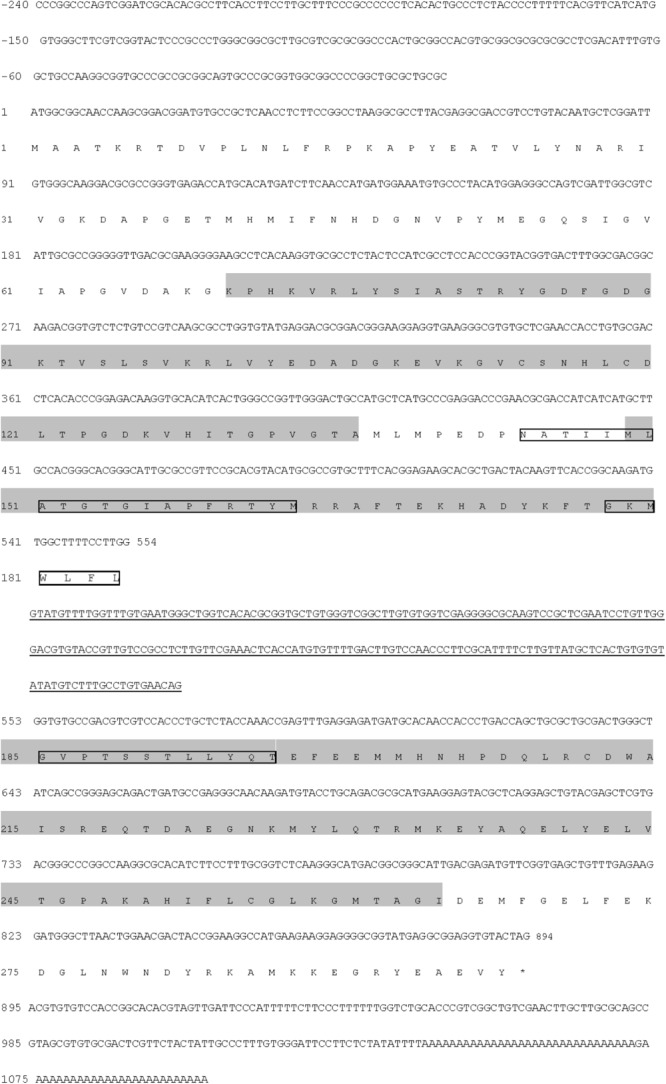
Nucleotide and deduced amino acid sequences of the *FNR* gene from *Py. yezoensis*. Nucleotides of the cDNA are numbered in the 5′ to 3′ direction. Amino acids are numbered in the N- to C-terminal direction. The underlined sequence is the intron. The black shading indicates the conserved FAD- and NAD(P)-binding domains. The asterisk represents the termination codon tag. The possible transmembrane regions are labeled with textboxes.

To investigate the molecular functions of FNR in the salinity stress response, the cDNA sequence of *Py. yezoensis* was cloned through RACE and Gene Walking technology. The full-length transcript, which has been submitted to GenBank under accession number HM370553, comprised 1339 nucleotides, including the poly (A)-tail. The sequence encoded a protein of 297 amino acids (**Figure 5**). There was a 3′-untranslated region (UTR) of 205 bp downstream of the stop codon. However, the polyadenylation signal (AATAAA) was not found in the 3′-UTR. A genomic DNA fragment of 1545 bp was amplified, which was found to possess one intron (underlined in **Figure 5**) by comparison between the cDNA sequence and the genomic DNA fragment. When the genomic DNA sequence was submitted to the NetGene2 Server, a potential intron was predicted at the same sites as that identified by comparing the cDNA and DNA sequences. Primary structural analysis of the sequence showed that the molecular weight of the putative FNR was 33.33 kDa, with a theoretical isoelectric point (pI) of 6.61. The protein possessed one FAD-binding domain (at position 70–136), one oxidoreductase NAD-binding domain (at position 149–264), and an alkaline amino terminal extension polypeptide (pI: 10.04) was detected using the Protein Analysis System (**Figure 5**). Moreover, two possible transmembrane helices were detected. The core region sequence positions were from 144 to 163 and 196 to 178 (outside→inside; **Figure 5**).

**FIGURE 6:**
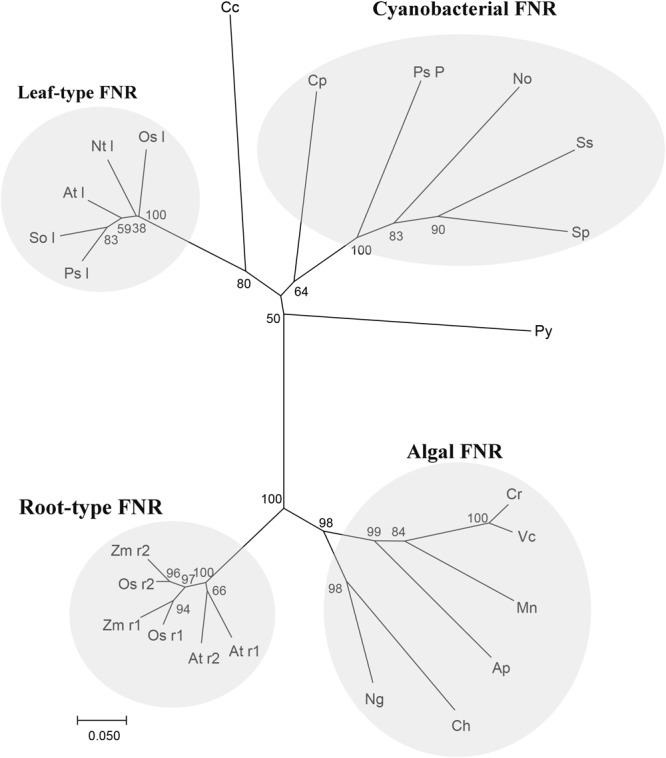
Relationships among FNR amino-acid sequences. Neighbor joining tree based on FNR protein sequence of Py (*Pyropia yezoensis*): ADM64306.2 and the other 23 species, including Cp (*Cyanophora paradoxa*): Q00598, Nt l (*Nicotiana tabacum leaf*): O04977, So l (*Spinacia oleracea leaf*): AAA34029, Sp (*Spirulina sp.*): P00454, Ss (*Synechocystis sp. PCC 6803*): CAA63961, Ps l (*Pisum sativum leaf*): ABO87610, Ap (*Auxenochlorella protothecoides*): XP_011400423.1, At l (*Arabidopsis thaliana leaf*): NP_201420.1, At r1 (*Arabidopsis thaliana* root1): NP_001190682.1, At r2 (*Arabidopsis thaliana* root2): NP_849734.1, Ch (*Chrysochromulina sp.*): KOO53489.1, Cr (*Chlamydomonas reinhardtii*): XP_001697352.1, Mn (*Monoraphidium neglectum*): XP_013905631.1, Ng (*Nannochloropsis gaditana*): XP_005853875.1, Os l(*Oryza sativa leaf*): BAS95764.1, Os r1 (*Oryza sativa root 1*): BAF13390.1, Os r2 (*Oryza sativa root 1)*:BAF20804.2, Ps P (*Pseudanabaena sp. PCC 7367*): WP_015165197.1, Vc (*Volvox carteri*): XP_002954986.1, Zm r1 (*Zea mays* root 1): ACG39703.1, Zm r2 (*Zea mays* root 2):ACG35047.1, Cc (*Cyanidium caldarium*): BAF42337.1 and No (*Nostocaceae*): BAO37114.1. Tree topology was inferred from maximum parsimony analyses, and the numbers at nodes were bootstrap support percentages of 1,000 replicates. Branch lengths were proportional to the level of sequence difference (note the scale bar). The four distinct branches are individually labeled on the right of the panel.

The alignment of *Py. yezoensis* FNR to those molecules deposited in the NCBI database revealed that the enzyme belonged to a CYPOR-like FNR, which catalyzed the reversible electron transfer between NADP(H) and Fd. The sequence of this molecule was ∼50% identical to most FNRs from other photosynthetic organisms. Based on the sequence information of *Py. yezoensis* and the other 23 species, which included most types of photosynthetic organisms, including higher plants, algae, and cyanobacteria, a phylogenetic tree was constructed, and the evolutionary relationships among the different FNRs were determined. As shown in **Figure 6**, the tree contained four discrete clusters (bootstrap ∼ 100%) corresponding to well-defined groups of organisms. The widely diverse leaf-type FNRs formed the first branch, and all cyanobacterial FNRs assembled into one group with relatively low bootstrap percentages. FNRs from the unicellular algae formed the third clade with tight clustering, and higher plant root FNRs were on another individual branch. Notably, the *Py. yezoensis* FNR did not belong to any already defined monophyletic group. However, this FNR showed a closer evolutionary relationship with the cyanobacterial FNR than with the leaf FNR.

### Variation of FNR Under Different Salinity Stress

**FIGURE 7:**
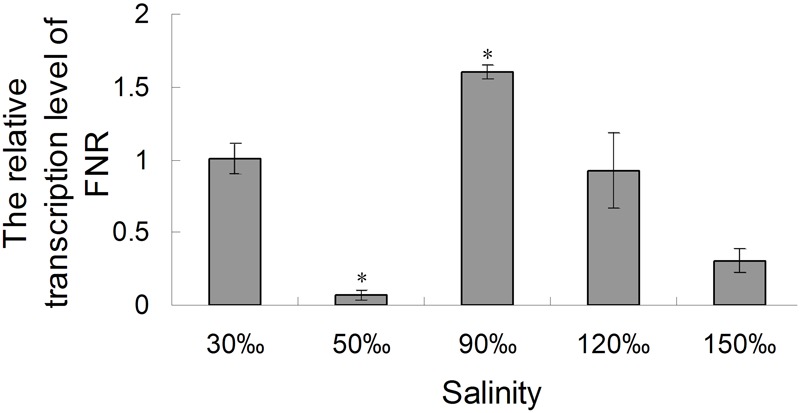
Real-time analysis of the relative expression of the *FNR* gene in *Py. yezoensis* under different salinities. A gene fragment from the *EF-1α* gene in the same sample was used as the internal control. The 2^-ΔΔ^*^C^*^t^ method was used to compare the relative fold changes in FNR, and the log2 of these variations was used to perform statistical analysis. Bars represent the mean values of three independent experiments ± SDs. ^∗^*P* < 0.05 compared with the control.

**FIGURE 8:**
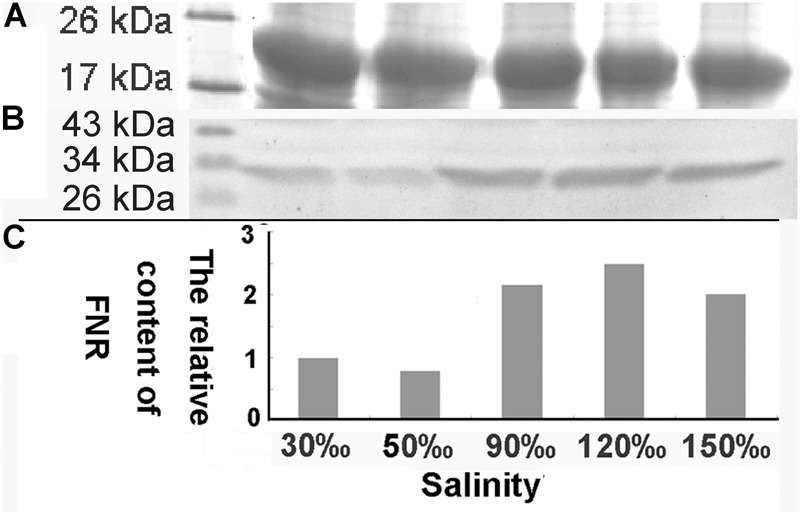
Western blot analysis of FNR in samples treated with different salinity stress. **(A)** Western blot analysis of samples treated with different salinities (Mark, 30, 50, 90, 120, and 150‰). **(B)** Equal proteins of corresponding samples were separated on SDS-PAGE and stained with Coomassie brilliant blue R-250. **(C)** The relative content of FNR derived from the normalization of western blot band by the 19 kDa protein band in SDS-PAGE gel.

The relative expression of the *FNR* gene decreased significantly when the salinity increased to 50‰ (0.069-fold compared with the control, *P* < 0.01), but increased at a salinity of 90‰ (1.596-fold compared with the control, *P* < 0.05). Its level then returned to baseline at 120‰ salinity and decreased again are the salinity increased further (**Figure 7**). Quantitative analysis (**Figure 8**) by western blotting revealed the similar variation profile as the real-time PCR. There was about twofold upregulation in FNR under salinity stress from 90 to 150‰ compared with that in the control. Slight downregulation (about 0.8-fold) was observed in samples treated with salinity 50‰.

## Discussion

### Determination of Different Degrees of Dehydration

Dehydration is one of the most important factors influencing the photosynthetic activity of intertidal algae (Davison and Pearson, 1996). Some studies have demonstrated the effects of dehydration on algae exposed to air directly. However, the exact water content in algae is hard to control during air-drying. Thus, some essential information on the dehydration response has been missing. Accordingly, in this study, we used increasing external salinity around algal tissues to obtain a dehydration series. The increased salt concentration withdrew water from cells via a water potential defined by the concentration of the ions in the external medium. This method had the advantage of maintaining specific water contents in the tissues and permitted greater reproducibility for replicate dehydrations compared with air-drying (Smith and Berry, 1986). In fact, *Py. yezoensis*, located in the high tide zone, usually experiences 1–2 h of desiccation with the tide cycle. Smith and Berry detected thresholds of photosynthetic tolerance in *P. perforate* using 3 M NaCl in seawater to simulate the range of dehydration encountered by *Pyropia* located in the intertidal zone (Smith and Berry, 1986). Thus, we used a 2-h salinity treatment to investigate electron transport in algae in response to high osmotic stress.

### Photosynthetic Parameters of *Py. yezoensis* Under Salinity Stress Conditions

The appropriate amount of actinic light applied during determination of photosynthetic parameters varies according to the individual species. Based on the results of rapid light curves, we found that salinity caused PS II susceptibility and thus decreased the maximal quantum yield (α) and the maximum ETR(II). However, the I_k_ values showed a slight increase when the algae were stressed with salinity below 90‰. This indicated that the fraction of light energy transfer from PS II to PS I increased with the stress response, consistent with the results reported previously (Fork and Öquist, 1981). At the same time, the results of rapid light curves with reliable actinic light (51 μmol photons m^-2^ s^-1^) were used to evaluate variations in the photosynthetic parameters of *Py. yezoensis* under high salinity treatments.

Salinity caused significant decreases in ETRs, suggesting that the photosynthetic states were affected profoundly by water loss. The suppression of PS II activity might be a mechanism to protect cells against over-reduction of the intersystem chain, which would result in the production of reactive oxygen species (Bukhov and Carpentier, 2004). The profile of ETR (I) indicated that the electron flow around PS I was elevated at 40‰ salinity, and this electron transport activity was still observed at 120‰ salinity, where the LEF was almost abolished. More importantly, the difference between ETR (I) and ETR (II) (*D*-value) of samples stressed with 40–120‰ salinity remained high, similar to that in the control, demonstrating that the fraction of CET around PS I had increased. These findings were consistent with the results of previous study (Satoh et al., 1983).

### Feasibility Analysis of Different CET Pathways Through Corresponding Inhibitor

Evidence for CET around PS I was also obtained by observation of variations in the P700^+^ signal under treatment with other special inhibitors. Antimycin A, a well-known inhibitor of respiratory cytochrome b/c1 complex, was defined as the effective inhibitor of pgr5-dependent CET (Tagawa et al., 1963). This compound blocked the reduction of PQ through cytochrome *b6* specifically. As an inhibitor of mitochondrial complex I, Rotenone was also reported to inhibit the activity of NDH-complex and thus the electron donation from NAD(P)H to PQ pool was restrained (Feild et al., 1998). In contrast, INN showed specific inhibition to both flavoproteins and the rotenone-insensitive NAD(P)H:(PQ-acceptor) oxidoreductase. The non-photochemical reduction of PQ pool was selectively suppressed by INN at the concentration of 250 μM whereas FNR activity was less affected (Corneille et al., 1998). This meant that we could use INN to investigate the CET pathway mediated with rotenone-insensitive NAD(P)H:(PQ-acceptor) oxidoreductase and no obvious interference from other metabolic reactions related to FNR was introduced.

### Involvement of Different CETs During High Salinity Stress Response of *Py. yezoensis*

Far-red light is preferentially absorbed by PS I and causes P700 oxidation, which does not result in full P700 oxidation due to CET around PS I. If the light intensity is sufficiently high to cause oxidation of P700 before CET or electrons from PS II can cause re-reduction of PS I, the maximum P700^+^ signal is recorded (Klughammer and Schreiber, 1994). Our findings showed that the immediate reduction of P700^+^ after shutting off the saturation pulse indicated that there existed certain CET which could maintain the PS I to be reductive in *Py. yezoensis* (**Figure 3**). The sudden onset of the saturating flash caused electrons to be expelled from PS I, as depicted by the steep increase in P700^+^ value, re-reduction was then observed (**Figure 3A**). Compared with the steep decrease in the P700^+^ signal in the control, severe salinity (e.g., 120‰) stress caused the suppression of PS II activity (**Figure 3A**), and the slower re-reduction of P700^+^ could be attributed to the shortage of electrons from PS II. While, once electron flow from PS II was inhibited by DCMU, the P700 presented the oxidation state upon the saturation pulse (**Figure 3B**). Unlike the stable P700^+^ signal in the control, the increasing trend in the P700^+^ signal attributed to the retardation of oxidation of PS I derived from the shortage of the electron acceptors under salinity stressed conditions (**Figure 3B**).

Under mild and moderate high salinity stresses, the electron donors contained in chloroplast could be transported to PS I under saturation pulse (**Figure 3A**). Inhibition of CET pathways by a certain inhibitor led to suspension of the corresponding electron transport from PS I to PQ. And thus, the limitation of electron acceptors of PS I caused a fraction of PS I unable to be oxidized, leading to the instantaneous reduction of the P700^+^ signal (**Figures 4A,B**). The changes of P700^+^ signal caused by AA, Ro, and INN showed similar patterns (**Figures 4A,B**). The observed effects of various inhibitors on the P700^+^ signal indicated that there were at least three different CET pathways working in the stress response of *Py. yezoensis*. Moreover, even the application together of these inhibitors did not suppress the electron transport form PS I to the electron acceptors, indicated by increase of P700^+^ signal under saturation pulse (**Figure 4**). Accordingly, a new or unidentified electron transport pathway may be still involved in the reduction of PS I. Further studies are needed to determine whether the postulated electron transport pathway in *Py. yezoensis* belongs to a kind of pseudo-CET (Shikanai and Yamamoto, 2017).

PS I-driven electron transport involves several different pathways, and each pathway is under the control of specific enzymes (Bukhov and Carpentier, 2004). Based on the near-infrared absorbance of PS I, which reflects the oxidation-reduction state of P700, Ravenel et al. (1994) revealed the coexistence of two different CET pathways in *C. reinhardtii*. One of these pathways was found to be mediated by Fd, and the other was found to be mediated by NAD(P)H (Ravenel et al., 1994). In *Py. yezoensis*, the diversity of CET pathways is related to the physiology importance of acclimation to the intertidal conditions. When the algae were subjected to dehydration stress, the Calvin cycle activity was reduced (Lawlor and Cornic, 2002). This led to the accumulation of reducing equivalents of NAD(P)H and reduced Fd derived from PS II. The PGR5-PGRL1 dependent CET pathway is believed to be an efficient, rapid-response mechanism that does not require *de novo* protein synthesis. It is particularly important in organisms that experience extreme environmental variations, such as macro-algae in the intertidal zone (Bukhov and Carpentier, 2004). With continuous increases in salinity, the ETR decreased as well (**Figure 2**), leading to major decreases in reduced Fd related to PS II (Zou and Gao, 2002). Thus, the supply of endogenous reductants may be a critical strategy for maintaining the function of CET around PS I. Notably, both NAD(P)H and NADH could donate electrons to reduce the PQ pool via the CET pathway mediated by NAD(P)H:(PQ-acceptor) oxidoreductase. Just due to the variety of CET pathways in *Py. yezoensis*, proper energization of the thylakoid membrane could be maintained to produce ATP to fuel active ion transportation (Jeanjean et al., 1993) under salinity stress conditions, or to supply the proper ATP/NAD(P)H ratio for synthesis of vitreous intercellular substances, such as glycerol, which was believed to be involved in metabolic regulation in osmotic alga (Ben-Amotz and Avron, 1973).

The variations in P700^+^ signal between different salinity treatments implied that the CET pathways and electron donors differed accordingly (**Figure 4**) in *Py. yezoensis*. And our results showed that these CETs were interlinked through the electron donor of NAD(P)H and reduced Fd. The continual increase after an instantaneous reduction of P700^+^ signal under the addition of a certain inhibitor (**Figures 4A,B**) revealed the involvement of other alternative CETs. The conversion of electron donors between reduced Fd and NAD(P)H may also be involved during the process. Under the severe dehydration in particular, the supply of reduced Fd from PS II was almost inhibited. Thus, the supply of reductive NAD(P)H from glycolysis was believed to be a very important endogenous electron resource. Ravenel et al. (1994) showed that the stromal components from starch degradation, as intermediates, seemed to play an important role in adaptation to environmental stresses in algae. Our previous results showed that prolonged dark treatment caused a decline in ETR (I) during dehydration in macro-algae (Gao and Wang, 2012). Which indicated that the NAD(P)H from starch degradation was equally important in supplying of electron donor in *Py. yezoensis* (Lu et al., 2016). Additionally, it was believed that the chloroplast NDH accepted electrons from NAD(P)H, which could be reduced by Fd via the reverse reaction mediated with FNR (Shikanai, 2015). On the other side, Iwai et al. (2010) isolated a protein supercomplex containing FNR engaged in the CET, but demonstrated that the supercomplex was sensitive to AA. Under the severe high salinity treatment in *Py. yezoensis*, contribution of the PGR5-PGRL1 protein-dependent pathway to the total CET was still important, as demonstrated by the obviously instant decrease in P700^+^ signal due to the closure of a certain PS I center (**Figure 4C**). Thus, it was feasible that FNR was involved in the regulation of different CETs in *Py. yezoensis* when the algae were subjected to high salinity stress treatments.

### Regulation of Electron Transfer by FNR in High Salinity Stressed *Py. yezoensis*

It is generally accepted that FNR is nuclear DNA encoded. The precursor protein was synthesized in the cytoplasm and transported into the chloroplast by a leader sequence in the N-terminus (Grossman et al., 1982). This transit sequence was then removed, and the mature protein was anchored in the chloroplast (Smeekens et al., 1990). We had constructed a low-coverage draft of *Py. yezoensis* genome using a Solexa high-throughput sequencing system and demonstrated that FNR was encoded in nuclear DNA (data not shown), although the leader peptides were not detected. The results of alignment between *Py. yezoensis* FNR and other FNRs deposited in the NCBI database revealed that the enzyme belonged to a CYPOR-like FNR, which could catalyze the electron transfer from NADP(H) to Fd^+^ (Hubbard et al., 2001). On the other hand, as illustrated in the phylogenetic tree, *Py. yezoensis* FNR was more closely related to that of cyanobacteria. Thus, the functionality of FNR in *Py. yezoensis* was similarity to that of cyanobacterial FNR, which was characterized by highly efficient electron transfer in both directions (Hanke et al., 2004). The N-terminal domain of FNR sequences obtained from a variety of cyanobacterial species was found to be similar to the CpcD polypeptide (Schluchter and Bryant, 1992). Moreover, the positively charged N-terminal sequence was believed to facilitate the association of FNR with phycobillin proteins, which were generally acidic, through electrostatic interactions (van Thor et al., 1998). As described above, there was an alkaline amino terminal extension polypeptide with an isoelectric point of 10.04 in *Py. yezoensis* FNR, suggesting that the protein discussed herein could transport electrons from NAD(P)H to oxidized Fd. The predicted transmembrane region of FNR suggested that the *Py. yezoensis* FNR could be embedded in the thylakoid membranes and bounded with the NDH complex by electrostatic force.

The original cyanobacteria FNR mediated both Fd-dependent NADP^+^ photoreduction and NAD(P)H-dependent Fd^+^ reduction under heterotrophic conditions. During the long evolution process, FNR had undergone multiple modifications to adapt to the metabolic challenges derived from the different lifestyles of their hosts (Ceccarelli et al., 2004). Hcp et al. (2002) reported that *de novo* synthesis of FNR was required to enhance CET in cyanobacteria. FNR was also proposed to be loosely bound to the chloroplastic NDH complex that reduced PQ from NAD(P)H in higher plants (Shikanai, 2014), several cyanobacteria (Mi et al., 1995), and algae (Seidelguyenot et al., 1996). In *C. reinhardtii*, the formation of a protein supercomplex composed of PS I, PS II, cytochrome b6f, and FNR was induced under stressed conditions and that this supercomplex was engaged in CET to alleviate stromal redox pressure (Mosebach et al., 2017). Moreover, the supercomplex showed sensitivity to AA, and cytochrome b_6_f was found to be reduced by NAD(P)H via FNR in an *in vitro* test. Our real-time PCR and western blotting results showed that the relative expression of FNR was upregulated under 90‰ and 120‰ salinity in *Py. yezoensis*. Additionally, the soluble sugar and G6PDH levels were increased under severe salinity stress in *Py. yezoensis* (Lu et al., 2016). Furthermore, 6-phosphogluconate dehydrogenase activity was upregulated with downregulation of cytosolic glyceraldehyde 3-phosphate dehydrogenase activity, reflecting upregulation of the oxidative pentose phosphate pathway (Lu et al., 2016). Thus, NAD(P)H from glycolysis is a critical endogenous electron donor in seaweed located in the intertidal zone. This led us to speculate that electrons may be transferred from FNR to Fd under the salinity stress conditions in *Py. yezoensis*. Here, through the connections between different donors bridged by FNR, electrons were channeled toward distinct routes to adapt to extreme variable environments. The hypothesis was consistent with our results of real-time PCR and western blotting.

As for the downregulation of FNR in the sample which was stressed with 50‰ salinity, one possible reason might be the feedback inhibition of FNR expression during mild high salinity stress. Since the CYPOR-like FNR, which was related to NAD(P)H cytochrome p450 reductases, is usually localized in chloroplasts, mitochondria, and bacteria, where it participates in a wide variety of redox metabolic pathways (Hubbard et al., 2001). Thus, the FNR of *Py. yezoensis* also participates in a number of other central pathways involving redox-based metabolisms. It was reported that the affinity of FNR to oxidized Fd should increase more than 10 fold on addition of NAD(P)H (Batie and Kamin, 1984). However, the Calvin cycle activity was reduced (Lawlor and Cornic, 2002) and nitrogen assimilation in the cytoplasm might be restrained under the stress conditions. This led to the accumulation of NAD(P)H and as a result the content of oxidized Fd would be maintained at a relatively low level. The shortage of substrates and the surplus of reducing equivalents caused downregulation of FNR.

## Conclusion

**FIGURE 9:**
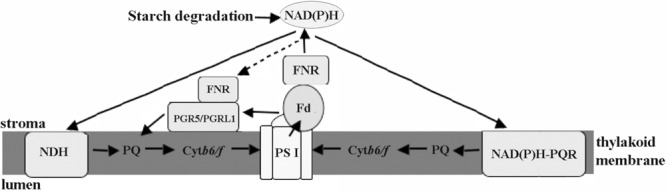
Schematic views of cyclic electron transport in *Py. yezoensis*. Three possible CET pathways around PSI were indicated by the arrows. The main CET pathway belonged to the PGR5/PGRL1-dependent CET using Fd as the electron donors. The other two pathways, NDH-dependent CET and the one mediated with rotenone-insensitive NAD(P)H-PQ oxidoreductase, were driven by NAD(P)H. FNR might be function as a regulator to maintain the proper contents of electron donors. PS I, photosystem I; Cytb6/f, cytochrome b6/f; Fd, Ferredoxin; FNR, ferredoxin NADP^+^ oxidoreductase; PQ, plastoquinone; NDH, NAD(P)H dehydrogenase; NAD(P)H-PQR, rotenone-insensitive NAD(P)H-PQ oxidoreductase. Black arrows show electron flows.

The chloroplastic components of eukaryotic algae located in the intertidal zone were directly involved in cell defense against stresses. Regulation of electron transport may be more important for eukaryotic intertidal seaweeds compared with the PS I-driven CET in higher plants. The results herein suggested that there were at least three different CET pathways in *Py. yezoensis*, including PGR5-PGRL1 protein-dependent pathway, NDH complex-dependent, and NAD(P)H:(PQ-acceptor) oxidoreductase-dependent pathways (**Figure 9**). And FNR appeared to operate in the conversion between Fd and NAD(P)H. Thus, under changing environmental conditions, the reducing power was channeled toward distinct routes in *Py. yezoensis*. Further studies of the metabolic pathways of this highly salt-tolerant organism will improve our understanding of the diversity and evolution of stress-response mechanisms in the plant kingdom.

## Author Contributions

JN and GW conceived and designed the research. BY, JN, JF, and MX conducted the experiments. XX, SG, and WG contributed new analytical tools. JN, BY, JF, and XX analyzed the data. JN and BY wrote the manuscript. All authors read and approved the manuscript.

## Conflict of Interest Statement

The authors declare that the research was conducted in the absence of any commercial or financial relationships that could be construed as a potential conflict of interest.

## Footnotes

1 http://www.cbs.dtu.dk/services/NetGene2/

2 http://www.ncbi.nlm.nih.gov/blast

3 http://www.expasy.org/

4 http://pfam.sanger.ac.uk/search

5 http://www.ch.embnet.org/software/TMPRED_form.html
